# Postnatal clubs for integrated postnatal care in Johannesburg, South Africa: a qualitative assessment of implementation

**DOI:** 10.1186/s12913-022-08684-x

**Published:** 2022-10-25

**Authors:** Ndinda Makina-Zimalirana, Jackie Dunlop, Anele Jiyane, Sophia Marie Bartels, Helen Struthers, James McIntyre, Kate Rees

**Affiliations:** 1 Anova Health Institute, Johannesburg, South Africa; 2 Division of Community Paediatrics, School of Public Health, University of Witwatersrand, Johannesburg, South Africa; 3 Department of Health Behaviour, University of North Carolina, Chapel Hill, USA; 4 Department of Medicine, Division of Infectious Diseases and HIV Medicine, University of Cape Town, Cape Town, South Africa; 5 School of Public Health and Family Medicine, University of Cape Town, Cape Town, South Africa; 6 Department of Community Health, School of Public Health, University of Witwatersrand, Johannesburg, South Africa

**Keywords:** Antiretroviral therapy, Differentiated service delivery, HIV, Health providers, Postnatal clubs, South Africa

## Abstract

**Background:**

South Africa has reported challenges in retaining women in Prevention of Mother-to-Child Transmission of HIV (PMTCT) programs postnatally. Due to the success of PMTCT in the antenatal period, proportionally more infant transmissions now occur after delivery. The Médecins sans Frontières (MSF) Postnatal Club (PNC) model allows for integrated postnatal care and support. Anova Health Institute implemented the model in primary health facilities in Johannesburg as part of a planned national scale-up. We aimed to assess the implementation of these PNCs.

**Methods:**

We used the RE-AIM (Reach, Adoption, Implementation, Maintenance) framework to assess implementation success and explore factors influencing implementation. In-depth interviews were conducted with 15 PNC staff, both clinicians and lay counsellors, using convenience sampling, from 12 facilities in Johannesburg. Data were analysed thematically using the RE-AIM framework.

**Results:**

PNC were perceived to have many benefits for postnatal clients and their infants: providers reported reduced waiting times, reduced number of clinic visits and that PNC provided clients with a space to form cohesive group dynamics thereby contributing to retention and adherence to antiretroviral therapy. However, it was found that lacking resources (e.g., space, medical equipment, staff) negatively impacted reach, implementation and sustainability. At times the PNC model was altered to accommodate the availability of resources (e.g., counselling mothers individually). Additionally, providers expressed concerns about lack of stakeholder adoption and emphasized the importance of involving facility leadership for successful integration of the model into routine primary healthcare.

**Conclusion:**

Our study found incomplete implementation of PNC in most of the participating facilities attributed to lack of resources and stakeholder buy-in. This underscores the need for increased support at management level to ensure sustainability. Effective collaboration between all stakeholders would allow better use of existing resources. Further studies are needed to evaluate whether all components of the model need to be implemented fully to ensure optimal outcomes, and to identify implementation strategies to facilitate scale-up.

**Supplementary Information:**

The online version contains supplementary material available at 10.1186/s12913-022-08684-x.

## Background

Antenatal Prevention of Mother-to-Child Transmission (PMTCT) services have been successfully implemented in South Africa, leading to a substantial reduction in vertical HIV transmission [1]. However, it has been difficult to retain women in PMTCT programs after they have given birth; with a review of large studies in a number of lower and middle countries, including South Africa, finding that 76% of pregnant women adhered to antiretroviral therapy (ART) during pregnancy, but only 53% did so post-birth [2] Overall, with fewer children becoming HIV-infected through vertical transmission, more than half of infant HIV-infections now occur in the postnatal period through breastfeeding rather than during pregnancy or labour [3]. To address postnatal PMTCT, carefully designed interventions need to target retaining women in care postnatally. One way of approaching this would be to provide integrated care to the mother-infant pair (MIP). There is a broad consensus on the value of integrating HIV and reproductive health services, particularly in regions of the world with generalised HIV/AIDS epidemics and high reproductive morbidity [4–6].

Postnatal Clubs (PNCs) are group based integrated, evidence-based model for comprehensive HIV and Maternal and Child Health care for postpartum women living with HIV and their HIV-exposed infants [7]. Previous research on group antenatal care interventions which have similar dynamics to PNCs have found high rates of improved quality of care [8–10], clients forming supportive relationships and open communication [10], and increased confidence in health decision-making and uptake [11]. Little is published on group-based postnatal care models in low- and middle-income countries [12] although Group Care Global is conducting implementation research on group antennal care and group care extended through two years postpartum as the 1000 Days model in South Africa [13].

Qualitative results from pilot program suggest that PNC are acceptable and feasible and trigger knowledge acquisition, behaviour change and offer peer support [14, 15]. Quantitatively PNCs show improved maternal viral load completion and infant testing uptake compared to historical controls [16].

However, translating this success to scale requires effective implementation of the model to achieve health impact. The shift from providing vertical services to the integration of postnatal HIV treatment services into maternal, neonatal and child health care is a complex transformation that demands significant adjustments in major aspects of health care organisation including providers’ workload [17], and infrastructural and logistic considerations (local service policies, physical space, information systems, equipment, drugs, and other medical supplies) [6, 17]. These factors can be analytically studied through Implementation Science (IS), which is the scientific practice of identifying, testing and scaling up effective interventions with high quality, fidelity and efficiency [18, 19].

Implementation Science is increasingly relevant within health programs to ensure that innovations and tools reach populations in need and are appropriately adapted to the local context while maintaining core elements of proven strategies. Implementation Science frameworks like the Reach, Effectiveness, Adoption, Implementation, and Maintenance (RE-AIM) have been established to guide interventionists to identify program components that will ‘‘improve the sustainable adoption and implementation of effective, evidence-based health promotion programs” [20]. Holtrop 2018 et al. demonstrated the value of qualitative measures in RE-AIM (and other planning and evaluation approaches) to address questions regarding the scalability and sustainability of innovations [21]. The paper demonstrated that qualitative methods help us understand how and why results on various individual RE-AIM dimensions, or patterns of results across dimensions (e.g., high reach and low effectiveness) occur.

The RE-AIM was chosen to evaluate PNC implementation because RE-AIM dimensions allow for the investigation of the degree to which an intervention can be adopted consistently and delivered in a sustained manner, reach large numbers of people and produce replicable behaviour changes [22] RE-AIM is a widely accepted framework used to assess the feasibility, quality, and public health impact of a health intervention [20] Additionally, the framework offers flexibility to address different public health concerns in a practical manner understandable by practitioners and policy makers [21].

While the PNC model has been adopted nationally in South Africa, it is yet to be implemented at scale. As a result, there is a need to understand how the PNC model is implemented to inform scale up to achieve the intended impact. To this end, we conducted a qualitative assessment of the implementation of postnatal clubs supported by the Anova Accelerating Programme Achievement to Control the Epidemic (APACE) program in Johannesburg. We utilised the RE-AIM framework for this study to provide an understanding of a range of factors influencing implementation outcomes.

## Methods

**Fig. 1:**
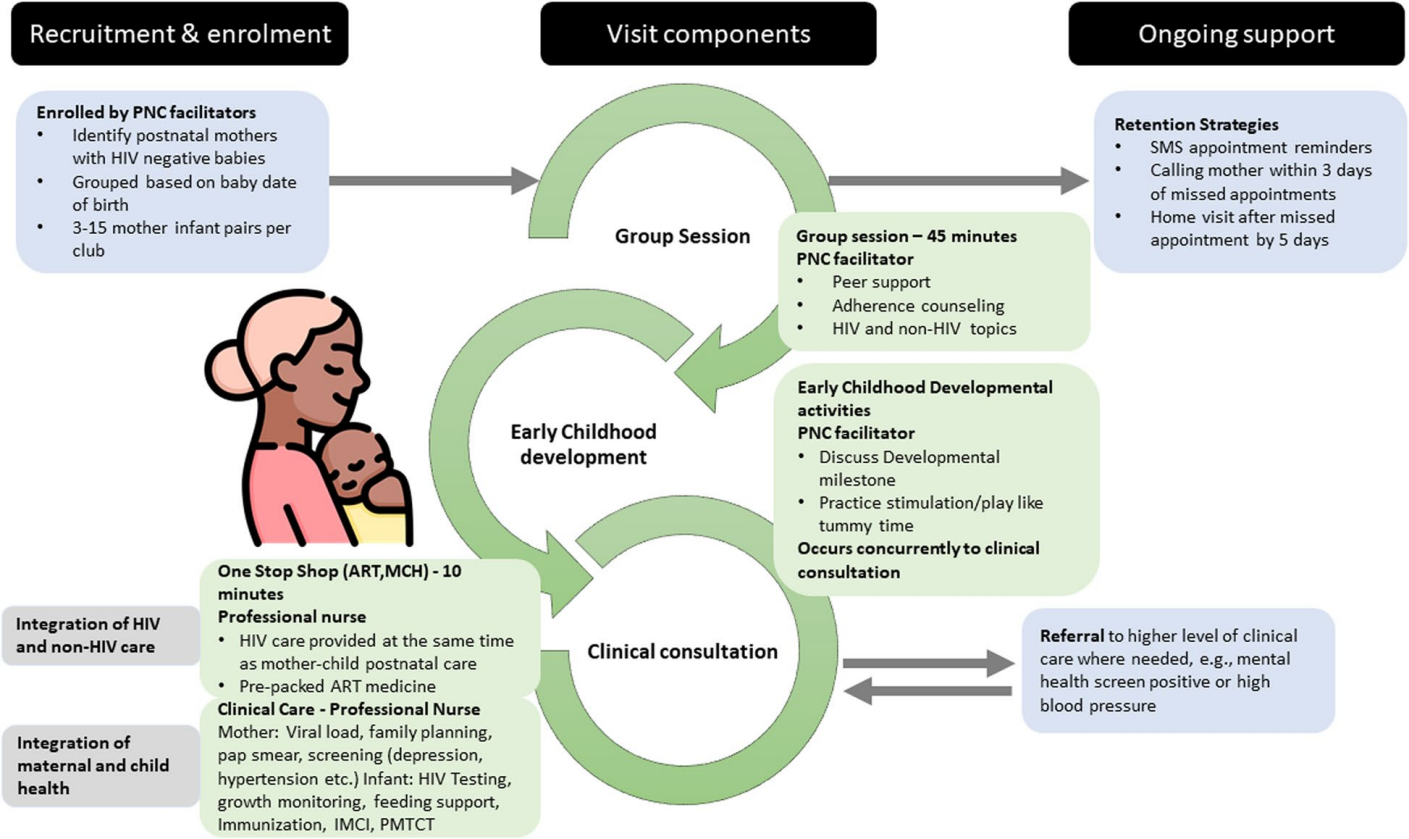
PNC components

PNCs (Fig. 1) were piloted in Khayelitsha by the City of Cape Town Health Department, Médecins Sans Frontières (MSF) and mothers2mothers (m2m) in June 2016. The theory of change below summarizes the pathway of change this intervention undertakes to ultimately contribute to improvements in maternal and infant health. The model’s theory of change is informed by the MSF tool kit [7] and WHO Framework on quality of maternal and new-born care in health facilities which defines the key components of quality hospital services for the mother and the new-born [23]. The WHO framework highlights two key aspects of quality: provision of care which includes evidence-based practices, efficient information, and referral systems and experience of car which includes effective communication, respect, dignity and emotional support [23]. The PNC model is built on the understanding that mitigating barriers to retention in care including long waiting times and high patient volumes at the antiretroviral (ARV) clinic and postnatal clinic, non-disclosure of HIV status, travel costs, inadequate knowledge about postnatal MTCT and stigma, could improve mother infant pairs positive experience of care (convenience, reduced wait time, peer support/cohesive group dynamics).

The model offers a comprehensive package of care to postpartum women living with HIV and their HIV-exposed infant(s). It integrates PMTCT and general (Maternal and Child Health) MCH care during the postnatal period. The services provided includes mental health and educational components and is delivered to postpartum women and their infants during an 18-month postnatal period. Facility and community-based mentor mothers provide peer’s support and health education on PMTCT and Early Childhood Development (ECD). The four pillars of PNC (as shown in Fig. 1) are:

- 1) Adult ART adherence club model which includes group peer support and adherence counselling for the mothers, (i.e., as in the adult ART adherence club model, the PNC starts with a peer support session, which is led by a club facilitator).
- 2) Early childhood development activities including ECD activities like tummy time,
- 3) Integration of maternal and child health which encourages a comprehensive one-stop shop for the clinical care for mother and baby at each session including pre-packaged ART, and
- 4) Integration of HIV and non-HIV care, for both mother and baby.

**Fig. 2:**
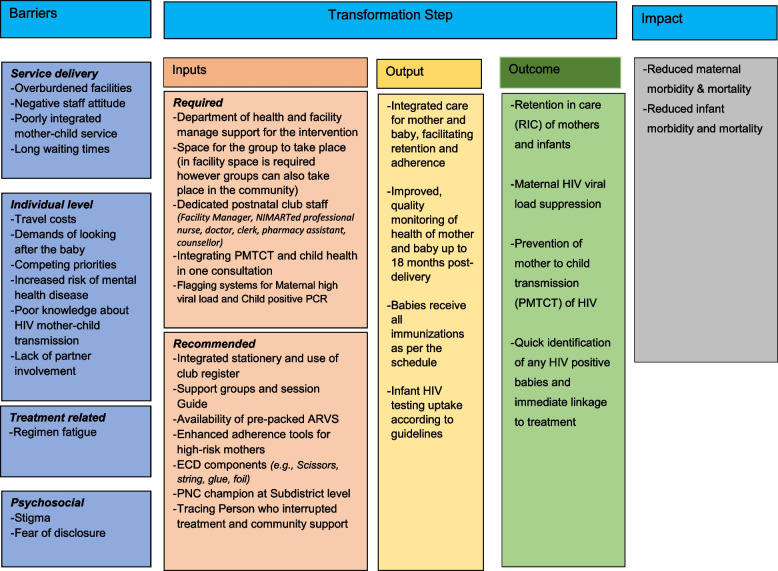
Theory of change

The model has previously been described in detail [7]. Figure 2 shows the models theory of change. In summary, during the HIV-positive mother’s first visit to the clinic following delivery (usually at 6 weeks), she is invited to join a PNC and given a date and time for the first session by PNC facilitator. A PNC consists of 3–15 MIPs and does not exceed the maximum number to ensure the emotional and psychological peer support and ECD activities can be carried out in an appropriate way. Any HIV positive mother regardless of age, VL and drug regimen can join a PNC with her HIV negative infant if she opts to. Babies born in the same month are grouped into clubs. In the first 6 months babies are seen monthly because of immunization schedule and their higher morbidity and mortality and risk in this period. After turning 6 months, clubs are held every three months until 18 months of age. These visits follow the “Road to Health” card clinical meetings: a health record in booklet form given to every new-born in South Africa which includes immunization cards, health messages, and other information relevant to early childhood development. PNC starts with a peer support session, which is led by a club facilitator followed by an integrated clinical session provided by the PNC nurse. Each visit’s interventions will depend on the age of the baby, and mother’s clinical care schedule is also tailored around the baby’s visits. The MIPs graduate from the PNC when the child reaches 18 months of age. At this stage, the mothers transition as a group into an adult club and the babies go to standard of care services. PNC facilitator is responsible for follow up within 2–3 day if the mother (or buddy) and baby do not attend a PNC session.

### Study setting

Johannesburg, one of 52 health districts in South Africa, has a population of about 5.5 million, almost 10% of the country’s population, and is the country’s largest Metropolitan District. The health district has over 600,000 people living with HIV (PLHIV), more than any other city worldwide [24]. Johannesburg is one of 27 health districts prioritised for HIV interventions by the US-funded President’s Emergency Plan for AIDS Relief (PEPFAR).

Anova is PEPFAR funded through USAID and a district support partner to the Department of Health (DoH) implementing HIV, tuberculosis and sexually transmitted infection programs in Johannesburg, Cape Town, Sedibeng, Mopani and Capricorn Districts. Anova supports the public health care system by providing technical assistance and direct service delivery. To implement PNC, Anova employed nurses and lay health providers to organise, recruit and run the clubs. All staff were trained on the PNC model using materials provided by MSF.

Anova and the Johannesburg Health District DoH established PNC at 12 public primary healthcare facilities in Johannesburg from July 2019 to June 2020. The study was conducted in all 12 of these facilities. By June 2020, there were 2300 MIPs enrolled in the PNC in the 12 facilities.

### Study design

We used semi-structured in-depth interviews (IDIs) to collect data from health care providers in Anova supported health facilities. We adapted semi-structured interview guides from the RE-AIM Qualitative Evaluation for Systematic Translation (RE-AIM QuEST mixed methods framework) [25]. The RE-AIM QuEST mixed methods framework recommends questions in each RE-AIM dimension that can be applied to a range of interventions, study types, and settings. Table 1 shows the RE-AIM dimension definitions and adapted key questions from which the study interview guides were developed.

**Table 1:** Implementation outcomes- RE-AIM dimensions and key questions about postnatal clubs addressed during data collection and analysis

RE-AIM Dimension	Definition	Key questions addressed
Reach	• Is the intervention reaching the target population?	➢ What are the barriers and facilitators to PNC enrolment, and how can they be addressed? ➢ What are the barriers and facilitators to participation for clients?
Effectiveness	• Does the intervention accomplish its goals?	➢ What are health providers’ perceptions on whether PNC is achieving the same patient outcomes and having the same impact as in the original design? ➢ What are the conditions and mechanisms that lead to effectiveness?
Adoption	• To what extent are those targeted to deliver the intervention participating?	➢ What affects health provider participation? ➢ What do health providers like about the intervention? ➢ What barriers and facilitators interacted with the intervention to prevent or encourage adoption? Was there partial or complete adoption? Why did some health providers in these facilities participate and others did not?
Implementation	• To what extent was the intervention consistently implemented	➢ What were the modifications to the intervention and why did they occur? ➢ What were the barriers to implementation? ➢ What are the contextual factors and processes’ underlying barriers to implementation and how do we address them?
Maintenance	• Extent to which the program has been institutionalised	➢ What are the barriers to continuing the program?

^*Note*: Adapted from RE−AIM QuEST mixed methods framework^

A researcher invited all PNC facilitating health providers, twenty in total, in the twelve PNC-implementing health facilities to participate in interviews. These health providers were specifically hired and trained by Anova to support DoH in PNC implementation.

We collected data in May and June 2020. A researcher (AJ) with experience in qualitative data collection conducted interviews telephonically using the semi-structured interview guides (Additional File 1). Interviews took between 40 to 80 min. All interviews were audio-recorded and later the voice recorded interviews were transcribed verbatim. Transcription was performed by the researcher who conducted the interviews. Transcripts were not returned to the participants for comment or correction.

### Analysis and interpretation

The electronic transcripts were loaded into NVivo 12 qualitative data software for analysis. We utilised a multistage analytical strategy to identify key themes, codes, and sub codes [26]. In the first stage, a researcher (NMZ) prepared an initial list of parent codes and definitions based on the study objectives, interview guide, and existing literature on group antenatal care. Examples of parent codes included: group sessions implementation, resource constraints, and psychosocial support and training. The same researcher applied these parent codes using four transcripts, in the process identifying more parent codes and relevant sub codes under each parent code. For example, under the group sessions implementation parent code we added the following sub codes: client-initiated discussions, topic guided discussions, group composition and confidentiality. In this way a preliminary codebook, which included both predetermined and emergent codes was developed. Then, all the remaining transcripts were coded using this preliminary codebook. Emergent codes that did not fit the defined codes above were added. Data were indexed by identifying segments of the data that corresponded to a particular code. Using thematic analysis [26], we analysed the coded transcripts using the key questions under each of the RE-AIM dimensions described in Table 1 above. All codes and themes were reviewed independently by another author (AJ).

### Ethical consideration

The Human Science Research Council (HSRC) research ethics committee (REC) approved the study, 3/22/08/18. All participants were above 18 years. We obtained informed written consent from participants before the beginning of the interviews. The research facilitator explained the purpose of the study prior to data collection. Participants consented to the use of a digital audio recorder. The transcripts and recordings were kept in password protected devices.

## Results

In total 15 interviews were conducted with Anova employed health care providers (9 lay health providers and 6 nurses). The health providers were interviewed based on availability and willingness to participate in the data collection. Interviews were conducted in 12 primary health facilities. The other 5 were not available because were either on leave or because they had other commitments during the time of data collection.

To provide context to the findings, respondents were asked to describe the duties which they conducted as part of their roles. The majority of the lay health providers described their role as that of a PNC facilitator, indicating that they are responsible for 1) preparing for PNCs and 2) conducting club sessions. Preparation of PNCs included recruiting MIPs, preparing meeting and consultation rooms, and ordering medication. During sessions they provided psychosocial support, conduct peer support, record club visits in the register, distribute pre-packed medication, and flag high viral loads and positive HIV test results to the PNC nurse. Outside of the PNC sessions, they trace flagged high viral load and positive HIV test clients.

The nurses reported that they were responsible for clinical oversight of PNCs. Activities included drug scripts for club members, provision of HIV and non-HIV clinical care for mothers and infants (e.g., general child health and PMTCT), blood tests (e.g., viral load, HIV tests), conducting pap smears and providing enhanced care for high-risk mothers.

Study participants reported that they provided services and identified a range of benefits, challenges, and suggestions; these are discussed below. The results are presented below within the dimensions of the RE-AIM framework.

### Reach

Reach refers to the number, proportion, and representativeness of individuals who are willing to participate in a given initiative, intervention, or program and can be assessed qualitatively based on individual’s perception of why clients accept or decline participation and describing characteristics of participants versus non-participants [21]. Key topics included enablers and barriers related to program enrolment and participation. When discussing recruitment strategies, the key facilitator was ‘‘One Stop Shop’’ model because several participants indicated using convenience services as a selling point when recruiting MIPS as illustrated by one participant,

“That it addresses everything that they will need and that it is going to be a one-stop-shop”

In turn, participants noted that the main reason clients declined to use the program was fear of unintended disclosure. They described how some clients avoid going to clinics nearest to them or participating in HIV care because they did not want to be seen at the clinic collecting ART medication or participating in adherence clubs and, in doing so, being linked to having an HIV positive status. Another reported barrier to enrolment was mothers’ perceived lack of importance of continued care postpartum. This was particularly seen in multiparous women.

Regarding participation, health providers cited that the model was preferable to clients, because it offered convenient medication pick-up processes and reduced waiting times and clinic visits. Furthermore, participants indicated that the model supports HIV status disclosure to families and provides clients with a space to form cohesive and positive group dynamics over time.

“They do like the model because they have built relationships with everyone, so we engage together, they like the whole setting of sitting together and seeing each other on the same appointment day”

PNC counsellors and nurses also cited that they were able to support clients beyond club days. Providers reported communicating through WhatsApp (either over groups or individually) which was also mentioned by providers as facilitating willingness of clients to participate in the model.

“And also, one other thing that they appreciate is this WhatsApp group that we have created because we can communicate with them to ask them to come to the clinic”

Time constraints to attend sessions was the primary barrier related to program participation. Participants explained that some of the mothers worked and led a busy lifestyle with regard to family, and social obligations.

“There are mothers who are working and as a result, they end up not forming the part of the club”

“Mother is working and now after the 4 months of maternity leave, she has to go back to work, and she will not be able to come to attend the session as they are supposed to”

### Effectiveness

Effectiveness focuses on important clinical or behavioral outcomes (in our case retention in care and adherence of ART) of interest and is most frequently summarized quantitatively [21]. However qualitative assessment is nonetheless important to provide insights on participants’ perceptions on how the intervention fared on its intended outcomes and any unintended outcomes and identify practical features that are contributors to program effectiveness. Our assessment indicated that providers perceived PNC attendance to be associated with a considerable increase in retention in care. Participants further indicated that the model was effective in improving client adherence to medication.

“Firstly, before we had the postnatal club, we used to have defaulters because they were waiting in the queue for a long time, waiting for medication and then the next day they have to come for family planning and then the following day they have to bring the child in for immunization and that takes a lot of time. After starting the Postnatal clubs, we see cooperation because the mother does not default anymore.”

“When we check the PCR register the were lots of babies who change their status from negative to positive but since this club came to the clinic the number of seroconversion babies is being reduced. They are not that much since the club came.”

Additionally, providers pointed out that PNC contributed to decongestion of health facilities, with MIPs able to receive ART care and child health services in a single clinic visit, decreasing the number of clinic consultations and visits that take place overall. Furthermore, participants indicated that the model provided enhanced care for MIPs.

“But now with the PNC, we dig deeper, we check the mother(‘s) viral load, we check if they are taking the medication, we engage with the mothers unlike when I used to do it before. PNC makes more sense than what I used to do before”

“I see the rate of testing has increased, so there was a gap for not testing well especially the 18 months”

Providers believed the model has a potential in improving retention in care and viral suppression, however, many participants stated that availability of resources (e.g., necessary clinical staff, space for sessions) affected proper PNC implementation ultimately determined the effectiveness of the intervention.

### Adoption

Adoption refers to number, proportion, and representativeness of settings and intervention agents (people who deliver the program) who are willing to initiate a program. The qualitative key issues in adoption parallel those of reach but are at levels of settings and staff/implementers and aims to understand why different organizations (in this case health facilities)—and staff members within these organizations—choose to participate or not; and to understand complex or subtle differences in those organizations and staff members in terms of underlying dynamics and processes [21]. Three themes emerged: 1) perceptions of value of the model; 2) quality of training and preparation; and 3) facility staff support.

Because all our study participants were specifically hired to implement PNCs, adoption at providers level was not an issue as in other interventions that rely on provider participation. All participating providers expressed support for the PNC model and a belief that the program has potential to provide efficient, comprehensive, and caring services. The assessment found consensus among participants that efforts towards scaling up PNC were perceived as worthwhile. Participants indicated the conventional PMTCT postnatal services, where mothers receive care independently from their infants, and HIV care may also be provided separately, do not cater to all maternal and infant needs. In their view, PNC was designed to address the specific needs and expectations of MIPs.

To ensure that the PNC model was implemented in a standardized format across the district, all Anova staff who were hired to work on PNCs received standardized training on the model and were given manuals and job aides. A majority of the participants indicated that they had received this training and that it assisted them with implementing the model.

“The workshop that I have mentioned, helped us a lot in a sense that by the time we got to the facility we were prepared with, and we knew what is expected from us and what we are dealing with and how to implement it.”

At facility level there was a lack of support from management and non-PNC implementing staff. Some participants indicated lack of collaboration from facility administrations affecting timely implementation, stating that it was difficult to convince facility management of the intervention’s value, which, in turn, delayed the process of obtaining the necessary resources (e.g., space) in time. This suggests that while adoption was universal among PNC direct implementers, it was lower among facility management who play a key role in implementation.

Additionally, most participants noted a lack of appreciation of facility staff in the model, with them not directly implementing postnatal clubs and questioning participants about the value of the PNC model. They reported that facility staff perceive that the intervention was developed for and implemented by an external entity.

“Because when you come in, they would say “no this is an Anova thing”

Despite facility staff’s lack of involvement, some participants reported pockets of support.

“Firstly, the management gave us all the support that we needed in terms of using their staff to attend to our patients on Fridays and again when we got here the two mentor mothers walked us through what happens on Fridays to conduct the club's because they had their experience …. on how to run the club's so they took us to step by step from getting files and preparing”

Another participant reported collaboration with other non-PNC implementing staff:

“Because we understand each other, if she sees someone even on her side (workstation) who is not the part of the club but needs the intervention, she knows where to direct that person and she knows how to assist that person and she knows what I am here for.”

Participants cited that involvement of government department stakeholders was necessary to promote their buy-in and support the integration of the intervention within established workflows of the clinics.

Participants suggested that demonstrating the competitive advantages of PNC with improved communication and dissemination would be beneficial, i.e., showcasing that the model was designed to make a difference. Participants emphasized the need to support other facility staff not directly involved in implementing the clubs to understand what role or gap PNC is designed to fill.

### Implementation

Implementation can be assessed at facility and individual level. At facility level, implementation refers to the intervention agents’ fidelity to the various elements of an intervention’s protocol, including consistency of delivery as intended and the time and cost of the intervention while at individual level, it refers to providers’ use of the intervention strategies [21]. Fidelity is typically measured quantitatively by having delivery staff or observers complete checklists noting which intervention core components are delivered; however, our qualitative method explored implementation issues including understanding providers perceptions of the conditions under which consistency and inconsistency are occurring across staff, setting, time, and different components of the intervention delivery.

While the PNC model has proven to be acceptable to our study participants, the implementation of the model is not without challenges*.* Participants reported variations in implementation that they attributed largely to a lack of resources. Health workers identified challenges including insufficient space for holding PNC sessions, limited human resource, and equipment. The concern about space was repeated by several participants, this challenge was linked to deviation from the group model. As a result of insufficient space, some providers were forced to conduct individual health talks rather than the intended group talks during sessions.

“……. if we can have space where we will do the sessions. Because now we call this PNC, but we see mothers one by one, and they do not know that they need to be in a group with all the other mothers.”

Inadequate human resource was also identified as a constraint to effective delivery of PNC in the facilities. Health providers reported that PNC are inadequately staffed, emphasizing the shortage of professional nurses, which resulted in nurses rotating among different facilities rather than being able to focus on PNC implementation at a single site. This had led to some facilities deviating from the model by conducting groups with different infant age categories in a single group. In the original PNC model, MIPs are grouped according to the age of the baby.

Other providers felt that appropriate planning prior to implementation should have taken place to ensure the necessary resources (e.g., space, staffing and equipment) were in place prior to the start of the project.

“I think before the Postnatal club was implemented in September; it would have worked if we identified the space where the session will take place. Because what happened is that we went to the facility, we told them about the PNC and we started and no resources were identified for us so, we had to arrange for ourselves to see how we go about.”

Other factors that constrained the implementation of PNC included insufficient medical equipment and lack of phones for communication with PNC participants. Participants reported that access to a “one-stop-shop” model was not possible in some facilities because of a lack of essential equipment, as equipment are shared among departments or programs, which meant clients had to move around the facility to access other services that cannot be provided in the same room.

“The baby scale [is a challenge] and I also struggle with the BP scale [blood pressure monitor] to check the blood pressure for the mothers. I do not have a weight scale. Sometimes it gets confusing for them because I tell them that we will do everything in one room, now I don’t do that. so, they need to go on the other side to do their weight and come back to me, so, those are my challenges.”

There were concerns around suboptimal implementation of clubs with some PNC components missing. Specifically, participants reported that there were no health talks, very few group meetings and clients were not accessing care in one room. This was cited by some providers to have contributed to a lack of adoption, likely because the model appears more cumbersome and less efficient than it was originally designed to be. Participants pointed out that the current state of implementation in some facilities was perceived by other staff as a duplication of existing immunization and PMTCT programs.

“So, there is no space to sit together and have a session so we both do it individually, so that is why I am saying it is a duplicate [of] what is being done on the other side of the clinic…… if I invite the sister to come and join us for the session, she would ask me what are you doing there that is different from what I do? and I would not be able to answer her because there is nothing that is different, so I do not see that there is a gap that is being filled.”

Participants reported challenges in the PNC implementation and indicated that they had to adapt the model to integrate with the health facility's processes. This flexibility is necessary as PNCs need to integrate with the health facility’s processes to be successful. This emphasizes the importance of maintaining agility when implementing PNC at health facilities.

### Maintenance

Maintenance refers to program sustainability (the extent to which a newly implemented intervention is “maintained or institutionalized within a service setting’s ongoing, stable operations) and the reasons why a) individual benefits continue or fade, and b) why the organization delivering the intervention decides to continue or discontinue the intervention [21]. The integration or institutionalisation of an intervention into routine practice has been linked with prospects for maintaining the intervention [21, 27]. A key driver of successful institutionalization at the facility level was having the necessary resources readily stocked and available, which requires support from and coordination with facility management. Participants believed that the project would likely not be sustainable in the future due to the lack of support and adoption of the model by the non-PNC focused facility staff.

That is why there is no support at all because they feel that its …… not a national mandate.

When asked what would facilitate continuing the project, participants felt that this will require increased advocacy support from facility management.

## Discussion and recommendations

The goal of this qualitative study was to understand factors influencing PNC implementation from health providers’ perspectives. Health providers generally perceived PNC to have many benefits to mothers and their infants; however, they felt that a lack of resources (e.g., space, medical equipment, staff) and lack of facility staff involvement affected implementation of the original PNC model and maintenance. Study participants reported variable implementation of PNC components due to the lack of resources, for example mothers were counselled individually instead of in groups.

Our findings suggest that participants perceived benefits of the intervention were selling points when recruiting potential clients, consequently facilitating intervention reach. This study found that the PNC model is perceived by participants to have the potential to enhance maternal retention in care and adherence to ART and prevent MTCT in the postnatal period. Results revealed barriers to reach, for example some clients believed additional support during the postnatal period is unnecessary. Other clients were reported to recognize the benefits of the model but encountered a range of barriers to participation including fear of unintended disclosure. An implementation science systematic review by Mukubanga et al. on barriers and facilitators to enrolment and participation in adherence clubs (from which PNC is adapted) identified similar barriers to reach or enrolment [28].

Our study described similar perceptions of effectiveness as previous studies on implementation of adherence clubs and postnatal groups [29, 30]. Adherence clubs were highly acceptable to stakeholders, given the observed benefits, including decongestion of clinics, increased social support for clients and the low cost of implementation [30, 31]. An MSF Project report showed that PNC have good early retention in care of the MIP and viral load suppression, as well as zero HIV seroconversion in the infants in similar setting [32]. Another study showed that the PNC model supports knowledge acquisition, triggers behavioural change, allows for peer support and satisfaction from the perspective of PNC clients, staff and key informants [15].

PNC were acceptable to PNC facilitating staff; however, the model is not well adopted by other non-PNC dedicated staff, and the management of the clubs can also be challenging. Our findings indicate that several facility-level factors hindered greater adoption of the intervention, including limited physical space for program delivery and low ownership by management and non-PNC staff who perceived the model to be duplication of the current standard of care. Adoption depends on the commitment of facility health teams and their understanding of the benefits of the model [17]. Many participants suggested increasing communication between stakeholders and fostering a culture of learning. As previously reported, communication can highlight positive experiences of providers and encourage clients to advocate for an intervention [33].

In general, we found inconsistent implementation of the four components of the model attributed to lack of resources highlighting the need to further interrogate the model to identify key components or combinations of components that could lead to similar benefits in terms of retention and viral suppression. The PNC model, which is based on the adult adherence club model, does allow for adaptions to different contexts, usually based on existing resources. A process evaluation of adherence clubs reported that different regions in South Africa vary in their approaches to implementing adherence clubs [28]. PNC have certain core components for the purpose of providing a structured program of implementation. For example, PNC requires that providers conduct group meetings and offer some level of “one-stop-shop” care to clients. Our study has shown that most facilities lack space and other resources, which made “one-stop-shop” care difficult and sometimes impossible. In these facilities, clubs would run without the group support component or care may be provided in different rooms of the clinic. It is not yet known whether PNC without the group or “one-stop-shop” components would lead to similar benefits in terms of retention and viral suppression in mothers. Without measuring fidelity or reporting on outcomes, it's unclear how adapting the model or dropping components affects the effectiveness of the intervention. While an adaption of the “one-stop-shop” may mean that all care is not provided in the same room, it may encourage better clinician communication, a reduction in waiting in queues at each service point, and lead to all care happening on the same day. Further evaluations could also measure fidelity to the original model which would help understand which components were essential for acceptability and effectiveness.

Sustainability is a limitation of many programs [6, 34]. Previous assessments of the model reported concerns around sustainability due to the perception that it increased the workload for staff involved. Similar to our results, stakeholders relevant to implementation of the model into real world practice reported that the expansion of the PNC model could be jeopardized by human resource constraints attributed to strong dependency on externally funded staff and minimal involvement of non-PNC focused staff [15]. Lessons learned from other differentiated models of care provide suggestions on ways to ensure sustainability of the PNCs, including task shifting of some tasks, such as counselling, drug dispensing and administrative tasks [28], which may be delegated to PNC graduates or PNC members.

Use of the RE-AIM framework allowed us to systematically identify facilitators, challenges, opportunities, and lessons learned, as related to reach, adoption, and sustainability, to be used in further implementation of PNC. While there has been a shift towards the integration of ART into routine post-delivery care, including differentiated care [4, 35] PNCs are yet to be scaled up in SA. Provider experiences from this study demonstrate that successful integration requires a health system-wide commitment at both planning and implementation stages [6, 36–38]. The RE-AIM framework has been used to provide a systematic way to engage service providers to refine interventions to fit within the intervention context. For example, Hacket et al. used the implementation outcomes framework to assess the implementation of a Postpartum Contraception Initiative in Tanzania to enhance the understanding of the implementation process, revealing the implementation dynamics, and highlighting potential entry points for improvement in program delivery [39]. The study found that providers were enthusiastic and receptive to the initiative. Similar to our study, they found that health system and resource constraints made adoption and implementation of the intended intervention challenging. Parallel to our findings, many providers questioned the sustainability of the initiative, and most agreed that changes to the initiative’s design (e.g., improved staffing, and availability of resources) would strengthen future iterations.

Similarly, Forman and colleagues applied the RE-AIM QuEST framework for both real-time and retrospective evaluation in a pragmatic cluster randomized controlled trial of the Adherence and Intensification of Medications (AIM) [25]. Using the framework, the researchers expanded retrospective evaluation of effectiveness by examining why the intervention worked or failed to work and explained which components of the intervention may have been barriers. Similarly, the framework helped our study to identify the group component of PNC as an implementation barrier.

This qualitative study has helped highlight that both pre and post intervention implementation stages are important to the success of intervention. Table 2 below summarizes recommendations categorized in phases from planning to implementation.

**Table 2:** Recommendations for planning and implementation of PNC

Stage	Recommendation
Planning	• Support facility managers and existing staff to implement PNC, including facility staff who will collaborate with implementation • Assess and allocate available physical resources, such as consultation and group space
Pre-implementation	• Training and sensitization of the implementing and other staff who collaborate in the implementation is important
Implementation	• Increasing communication between stakeholders and fostering a culture of learning among stakeholders • Regular interactions with stakeholders throughout implementation to monitor and adapt the intervention as needed
Scale Up	• Further evaluation to explore if the model as it exists is optimal and efficient and whether all four components are needed to ensure similar outcomes. This could support identification of the core components of the intervention

This study had limitations. Data collection did not involve non-Anova health providers nor PNC clients, so the study team did not have the opportunity to triangulate the results with these two groups. These groups could have taught us more about the barriers and facilitators to reach, adoption and sustainability of the project.

## Conclusion

At this early stage of implementation, our study found incomplete implementation of PNC in many of the participating facilities, mostly due to a lack of resources and stakeholder involvement. This underscores the need for increased support at management level to ensure model efficacy and sustainability. The assessment also highlights the importance of exploring resource availability at the organisation level to tailor elements of the intervention to fit within available resources. This study has shown that the PNC model can be implemented with flexibility, however without measures of fidelity and effectiveness, it is unknown whether the desired outcomes are still achieved.

Lessons learned from our analysis indicate the need to improve scale up and integration using scaling-up frameworks; we planned an integration phase, which could potentially have been strengthened in terms of adoption, acceptability to non-PNC facility staff and sustainability, by using a framework like ExpandNet [40].

## Supplementary Information

**Additional file 1.** Supplementary Material for: Postnatal clubs for integrated postnatal care in Johannesburg, South Africa: a qualitative assessment of implementation. Interview Guide.

## Data Availability

The dataset (which includes individual transcripts) is not publicly available due to the confidential nature of interview data.

## Abbreviations

DMoC: Differentiated Modalities of Care
DoH: Department of Health
ART: Antiretroviral Therapy
ECD: Early Childhood Development
HSRC: Human Science Research Council
MCH: Maternal and Child Health
MIPS: Mother-Infant Pairs
MSF: Médecins Sans Frontières
PEPFAR: President’s Emergency Plan for AIDS Relief
PLHIV: People Living with HIV
PMTCT: Prevention of Mother-To-Child Transmission Of HIV
PNC: Postnatal Club
RE-AIM: Reach, Adoption, Implementation, Maintenance
RE-AIM QuEST: RE-AIM Qualitative Evaluation For Systematic Translation
REC: Research Ethics Committee
