# Supplementary material for: Postnatal clubs for integrated postnatal care in Johannesburg, South Africa: a qualitative assessment of implementation
Source: BMC Health Serv Res. 2022 Oct 25;22:1286. doi: 10.1186/s12913-022-08684-x (PMC9598026; doi:10.1186/s12913-022-08684-x)
Supplement: Supplementary file 1 — Additional file 1. Supplementary Material for: Postnatal clubs for integrated postnatal care in Johannesburg, South Africa: a qualitative assessment of implementation. Interview Guide. [file 12913_2022_8684_MOESM1_ESM.docx]

**Supplementary Material for: Postnatal clubs for integrated postnatal care in Johannesburg, South Africa: a qualitative assessment of implementation**

**Interview Guide**

**Introduction**

My name is----------------------------------------------------------- (Name of interviewer). You are invited to participate in an assessment on Post Natal Club (PNC) project. The assessment aims to understand the process of PNC implementation and contextual factors influencing implementation outcomes. As part of the assessment, we are talking to a wide cross section of Post Natal Clubs service providers and users. Through participation in the assessment, you can contribute to improvement of Post Natal Club services. If you choose to participate in the assessment, you will be asked to complete an informed consent form.

|  | | | | |
| --- | --- | --- | --- | --- |
| Region: |  | Interviewer |  | |
| Facility/Hospital: |  | Date: | dd/mm/yy | |
|  |  | Time: | From: | To: |
| Results Code |  |  |  |  |
| Completed | 1 |  | . |  |
| Incomplete | 2 |  |  |  |
| Refused | 4 |  |  |  |
| Other (Specify) | | | | |

1. **Can you briefly describe your current role?**
   1. **What is your role with regard to Postnatal clubs (PNC) activities?**
2. **What are the biggest problems with Prevention of mother-to-child transmission** (**PMTCT) and Antenatal Care (ANC) in the district right now?**
3. **What do you understand by PNC?**
   1. **What do you know about PNC project?**
   2. **What do you know about how PNC came about and how/ why they were created? Who was involved? What did you think of the process? Had you heard about PNC prior to them being introduced at your facility?**
4. **Have you received any training or orientation regarding PNC?**
   1. **Who was involved in the training/orientation?**
   2. **How would you describe your participation in the training/Orientation?**
   3. **Do you feel all appropriate stakeholders were involved in orientation or training?**
5. **Can you tell me about the PNC project in this facility?**
   1. **How would you describe PNC integration with other mother and child health activities?**
   2. **How important are Postnatal clubs in the facility?**
      1. **How important is postnatal care?**
      2. **What are the gaps in postnatal care?**
      3. **Do you feel PNC met a need? How?**
   3. **How important do you think Postnatal clubs are to the community?**
   4. **What would you say would improve the PNC-related health service providers in performing their role in the facility?**
   5. **Do you think PNC taking place in the community is a viable option? Why?**
6. **What has been your experience with PNC program?**
   1. **Does the program fit well with normal practice of the facility?**
   2. **Does the program fit well with clients normal practice or expectation?**
7. **Can you describe how you support in the planning and execution of the PNC program?**
   1. **What are the challenges you face in planning? (Probe: Staffing, equipment, space, time taken to provide care)**
   2. **What are the challenges you face in providing support to PNC team? (Probe: Staffing, equipment, space, time taken to provide care)**
8. **Can you describe other challenges in delivering the PNC program?? (Probe: accessibility, availability of drugs, space, equipment and others)**
9. **What do you think should be improved with regard to delivering PNC program at the facility level?**
10. **What kind of activities/measures would further support in strengthening the delivery of PNC project?**
11. **How do you envisage PNC continuing in your facility in the next year?**
    1. **When Anova is no longer able to facilitate PNC in your facility, how do you see the future for PNC?**
12. **Is there anything else you would like to tell us about your experience with PNC?**
